# Prevalence and associated risk factors for intimate partner violence (IPV) in the Himalayan mountain villages of Pakistan

**DOI:** 10.1192/j.eurpsy.2022.811

**Published:** 2022-09-01

**Authors:** S. Yunus, S. Shah, G. Noshad

**Affiliations:** 1 UAE University, College Of Medicine And Health Sciences, Public Health Institute, Al Ain, United Arab Emirates; 2 University of Massachusetts, Center For Clinical Research, Worcester, United States of America

**Keywords:** Intimate Partner Violence, married women, Ghizar, Pakistan, Depression

## Abstract

**Introduction:**

Intimate partner Violence (IPV) against women includes all actions that violate one’s sense of self, physical body and sense of trust and involves episodes of violence of physical, psychological (emotional), or sexual nature, perpetrated by a current or former intimate partner.

**Objectives:**

We estimated the prevalence of and risk factors for intimate partner violence (IPV) in the Himalayan mountain villages of Gilgit Baltistan in Pakistan.

**Methods:**

We employed a cross-sectional study to randomly select ever married women (n=789) aged 18-49, in Pakistan. We used an adapted World Health Organization screening instrument to assess women’s experience of IPV in the previous 12 months. We used an indigenous validated instrument assess self-reported symptoms of major depression according to the DSM IV. Multivariable logistic regression analysis was used to identify significant predictors of IPV using adjusted odds ratio (AOR) with 95% confidence intervals (CI).

**Results:**

The overall prevalence of IPV was 22.8% (95% CI: 20.0-25.9). Women exposed to IPV were less likely to have husbands educated at a college or a higher (AOR: 0.40; 95%CI: 0.22-0.70), household income in the middle or the highest tertile (AOR: 0.44; 95%CI: 0.29-0.68), and were more likely to have poor or very poor relationship with their mother in law (AOR=2.85; 95% CI: 1.90-4.28), to have a poor quality of health (AOR= 2.74; 95% CI: 1.92-3.92) poor quality of life (AOR= 3.54; 95%CI: 1.90-6.58), and higher odds of experiencing depressive symptoms (AOR=1.97; 95%CI:1.39-2.77).

**Conclusions:**

IPV is a substantial public health burden in Himalayan mountain villages and merits serious attention.

**Disclosure:**

No significant relationships.

